# Math Anxiety in Combination With Low Visuospatial Memory Impairs Math Learning in Children

**DOI:** 10.3389/fpsyg.2019.00089

**Published:** 2019-01-31

**Authors:** Mojtaba Soltanlou, Christina Artemenko, Thomas Dresler, Andreas J. Fallgatter, Ann-Christine Ehlis, Hans-Christoph Nuerk

**Affiliations:** ^1^Department of Psychology, University of Tuebingen, Tübingen, Germany; ^2^LEAD Graduate School & Research Network, University of Tuebingen, Tübingen, Germany; ^3^Leibniz-Institut für Wissensmedien, Tübingen, Germany; ^4^Department of Psychiatry and Psychotherapy, University Hospital of Tuebingen, Tübingen, Germany; ^5^Center for Integrative Neuroscience, Excellence Cluster, University of Tuebingen, Tübingen, Germany

**Keywords:** math anxiety, math learning, children, individual differences, visuospatial working memory, processing efficiency theory

## Abstract

Math anxiety impairs academic achievements in mathematics. According to the processing efficiency theory (PET), the adverse effect is the result of reduced processing capacity in working memory (WM). However, this relationship has been examined mostly with correlational designs. Therefore, using an intervention paradigm, we examined the effects of math anxiety on math learning. Twenty-five 5th graders underwent seven training sessions of multiplication over the course of 2 weeks. Children were faster and made fewer errors in solving trained problems than untrained problems after learning. By testing the relationship between math anxiety, WM, and math learning, we found that if children have little or no math anxiety, enough WM resources are left for math learning, so learning is not impeded. If they have high math anxiety and high visuospatial WM, some WM resources are needed to deal with math anxiety but learning is still supported. However, if they have high math anxiety and low visuospatial WM capacity, math learning is significantly impaired. These children have less capacity to learn new math content as cognitive resources are diverted to deal with their math anxiety. We conclude that math anxiety not only hinders children’s performance in the present but potentially has long-lasting consequences, because it impairs not only math performance but also math learning. This intervention study partially supports the PET because only the combination of high math anxiety and low WM capacity seems critical for hindering math learning. Moreover, an adverse effect of math anxiety was observed on performance effectiveness (response accuracy) but not processing efficiency (response time).

## Introduction

Math acquisition is influenced by emotional factors such as math anxiety (Dowker et al., 2016). Individuals suffering from math anxiety experience a negative feeling whenever they are presented with mathematics, which impairs their math performance (Devine et al., 2012; Suarez-Pellicioni et al., 2016). Highly math-anxious individuals take a longer time to respond and/or make more errors than individuals with less math anxiety during math problem solving. Supporting the behavioral findings, neuroimaging studies have shown that math anxiety triggers the fear and hyper-sensitive brain network (for a review see Artemenko et al., 2015). This negative relation between math anxiety and math performance has been explained in different ways. Ashcraft (2002) suggests that highly math-anxious individuals tend to avoid activities and situations that require math. As a consequence, they have less practice with math, which hinders their math knowledge and ability. Another explanation is that highly math-anxious individuals who think that they are bad at math, can be easily distracted during the task (Eysenck et al., 2007) because they do not feel self-confident, and do not allocate their maximum effort to the task (Dowker et al., 2016).

In addition to emotional factors, cognitive processes such as working memory (WM) have been frequently shown to be core determinants for successful learning in school (e.g., Aronen et al., 2005; Lee and Bull, 2016). Lee and Bull (2016) argued that WM is needed while learning new skills including math and also to integrate the new information with previously acquired knowledge. According to Baddeley’s model (Baddeley, 1992), WM contains three components: (i) the visuospatial WM, known as the visuospatial sketchpad, which is a transient storage space for visual and spatial information; (ii) the verbal WM, known as the phonological loop, or the transient storage of verbal information; and (iii) the central executive, which is involved in regulating, manipulating, and generally processing the stored information. Prior studies have shown that different WM components play distinct roles in academic achievement during development. For instance, visuospatial WM was a strong predictor of math performance in 7- to 9-year-old children, whereas verbal WM and central executive were not (Holmes and Adams, 2006). Soltanlou et al. (2015) revealed that verbal WM was the best predictor of multiplication performance in grade 3 (8–11 years old); however, visuospatial WM was the best predictor of multiplication performance a year later in grade 4. In general, there is agreement that WM has an integral role in math performance (Menon, 2016; but see Nemati et al., 2017).

Working memory processes *per se* are also influenced by emotional factors such as math anxiety. The literature shows that math anxiety interferes with different WM components. For instance, Passolunghi et al. (2016) observed that children with low math anxiety show a better verbal WM than highly math-anxious children in grades 6 to 8 (11–15 years old). DeCaro et al. (2010) investigated the performance of adults on two kinds of verbal WM-based and visual WM-based math tasks during low- and high-pressure testing situations. The authors found that while a high-pressure situation attenuated the performance in a verbal WM-based math task, it was not influential in the visual WM-based task. They suggested that anxiety has a greater influence on verbal WM rather than visual WM. However, several other studies suggest a selective disruption effect of anxiety on visual WM in adults (Miller and Bichsel, 2004; Shackman et al., 2006) and in children in grades 1 and 2 (7–9 years old) (Vukovic et al., 2013). Despite these inconsistent findings across the literature, there is general agreement that anxious thoughts partially occupy WM capacities, which disrupts math performance.

**FIGURE 1:**
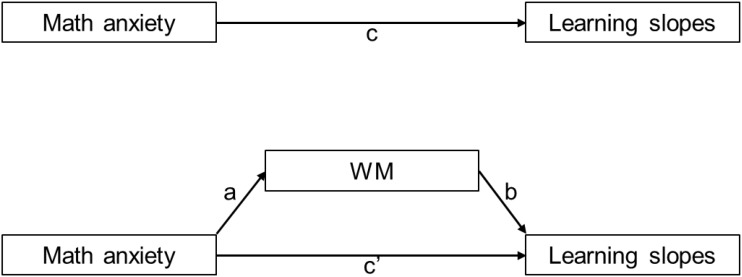
Path diagram: above panel depicts the total effect of the predictor (math anxiety) on the dependent variable (learning slopes) and below panel depicts the direct effect of the predictor on dependent variable while controlling for the mediator (WM components) and the indirect effect of the predictor on the dependent variable through the mediator.

As mentioned above, math anxiety, WM, and math performance are related to each other, whereby WM has been suggested to mediate the anxiety-performance relationship (cf. Figure 1). The processing efficiency theory (PET, Eysenck and Calvo, 1992) offers a good explanation for the interaction between them. The PET was developed based on Baddeley’s model of WM (Baddeley, 1992) and suggests that anxiety causes worry, which reduces the WM capacity, disrupting concurrent tasks. It contains two main concepts: performance effectiveness and processing efficiency (Eysenck and Calvo, 1992). Performance effectiveness refers to the quality of performance, i.e., the response accuracy, while processing efficiency refers to the relationship between performance effectiveness and a load of effort or cognitive resources, i.e., response time. For instance, occupying WM capacity leads to performance impairment (affecting performance effectiveness), but availability of auxiliary cognitive resources maintains a given performance level but at the cost of increased effort (affecting processing efficiency). Therefore, according to the PET, WM might be the best intermediate variable explaining the relationship between math anxiety and math performance.

There are two different accounts regarding the interaction of math anxiety, WM and math performance. One account is that individuals with higher WM capacity have more resources to simultaneously manage math anxiety and solve math problems (Ashcraft and Kirk, 2001). For example, a study in 11- to 12-year-old children reported that verbal WM accounts for 51% of the association between trait anxiety and academic performance including math (Owens et al., 2008). Therefore, children with low WM capacity suffer more from math anxiety during math problem solving. The other account suggests that individuals with higher WM capacity suffer more from math anxiety (Beilock and Carr, 2005) because they rely heavily on WM strategies to solve math problems. Therefore, under any high-pressure situation, their capacity is co-opted and they show a worse performance (Ramirez et al., 2013). This deficit does not occur for individuals with lower WM capacity because they do not rely massively on WM strategies to solve math problems in the first place, but rather use other strategies. Therefore, their performance does not drastically diminish in high-pressure situations. For instance, Ramirez et al. (2013) reported a relationship between math anxiety and verbal WM in children with higher WM capacity in grades 1 and 2 (see also Vukovic et al., 2013). So, despite contradictory findings across mediation studies, they mostly agree on the mediating role of WM in the association between math anxiety and math performance.

Although these relationships have been frequently studied, most of our knowledge comes from correlational studies, which have investigated the influence of math anxiety on a single measure of math performance. Therefore, longitudinal (e.g., Vukovic et al., 2013; Cargnelutti et al., 2016) and intervention studies are needed to clarify the causality of these relationships (Dowker et al., 2016). While correlational studies reveal possible associations between two variables, causal studies indicate the directionality of these associations. For instance, correlational studies revealed that math anxiety is associated with poor performance in both WM and math tasks. However, this relationship can be bidirectional: (i) math anxiety preoccupies WM and individuals attend less to the task (Eysenck et al., 2007), which leads to a low score on WM and math tasks (Ashcraft and Kirk, 2001), and (ii) poor math knowledge makes individuals worry because they feel incapable of solving math problems, so they show a high score on math anxiety tests (Maloney et al., 2011; Núñez-Peña and Suárez-Pellicioni, 2014; Lindskog et al., 2017). Therefore, the perennial “chicken and egg” question will not be resolved by correlational studies and intervention studies are needed (Dowker et al., 2016).

In one of the few longitudinal studies, Cargnelutti et al. (2016) observed that math anxiety and math performance have a bidirectional relationship. Nevertheless, math performance has a greater impact on math anxiety in 2nd graders (7–9 years old), whereas the reverse directionality was observed a year later in 3rd graders. Interestingly, they observed an indirect effect of math anxiety in 2nd graders on math performance in 3rd graders, suggesting poor math skills may cause math anxiety in younger children that disrupts math performance later. Supporting this finding, Ma and Xu (2004) suggested that prior math achievement longitudinally predicts later attitudes toward math across grades 7 to 12. However, the influence of WM on the association between math anxiety and performance was not investigated in these studies. Another longitudinal study (Vukovic et al., 2013) investigated this relationship by taking into account the WM capacity. The authors observed that high math anxiety in 2nd graders predicts less math acquisition from grade 2 to grade 3 but only in children with higher visuospatial WM capacity. Vukovic et al. (2013) suggested that math anxiety causes poor math learning by affecting WM resources in school children.

Longitudinal studies, however, also come with the possible confounding effects of brain maturation and concurrent economic trends or other events affecting children’s lives over a long timescale. Therefore, the findings of training studies might differ from longitudinal studies (Soltanlou et al., 2018). Accordingly, we conducted an intervention study in children to uncover the association between math anxiety and math learning, namely the difference in competence before and after learning. Furthermore, the possible mediating roles of different WM components were tested. We hypothesized that higher math anxiety leads to less benefit from arithmetic learning, and that this relationship is modulated by WM.

## Materials and Methods

### Participants

Twenty six typically developing children from 5th grade participated in the study. One child, who quitted training, was excluded and the remaining 25 children (9 girls; 11.13 ± 0.46 years old) were included in the analyses. All children were right-handed and had normal or corrected-to-normal vision with no history of neurological or mental disorders. Intellectual ability was measured by completing two subtests (similarities and matrix reasoning) of the German version of the Wechsler Intelligence Scale (Petermann et al., 2007), with resulting scores of 107.40 ± 11.65 and 107.80 ± 10.61, respectively. Children and their parents gave written informed consent and received an expense allowance for their participation. All procedures of the study were in line with the latest revision of the Declaration of Helsinki and were approved by the ethics committee of the University Hospital of Tuebingen.

### Material

#### Math Anxiety

Math anxiety was assessed by selected items from the German translation of the math anxiety questionnaire (MAQ) (Thomas and Dowker, 2000; Krinzinger et al., 2007), which has an internal consistency (Cronbach’s alpha) of 0.83–0.91 for the whole questionnaire for different age groups. In the questionnaire, we assessed three out of four subscales of the MAQ: self-assessment in math, attitude toward math, and concerns about math^1^. In our questionnaire, each subscale contains five items describing different math-related topics (calculation, handwritten calculation, mental calculation, simple calculation problems, and difficult calculation problems). While the subscales self-assessment in math and attitude toward math demonstrate general math-related attitudes, the subscale concerns about math indicates math anxiety (Krinzinger et al., 2009). Since we are only interested in the influence of math anxiety on math learning, we focus on the last subscale hereafter. This subscale includes five items, which are rated on a five-point Likert scale (ranging from 0 = very happy to 4 = very unhappy) with a maximum score of 20. Thereby, higher values indicate higher math anxiety.

#### Working Memory

Following Baddeley’s model (Baddeley, 1992), three components of WM, i.e., verbal WM, visuospatial WM, and central executive were measured. To this end, the letter span test (Soltanlou et al., 2015) and the Corsi block-tapping test (Corsi, 1973) were used. In the letter span test, the child had to recall spoken sequences of letters (presentation rate: one letter per second). The test was started with sequences of two letters. The sequence length was increased by one letter if the child recalled correctly at least one out of two sequences; otherwise, testing was stopped. In the Corsi block-tapping test, the child was asked to point to the cubes in the same order as the experimenter. Children started with sequences of three cubes. The sequence length was increased by one cube if the child recalled correctly at least two out of three sequences; otherwise, testing was stopped. For the backward in both tasks, children were asked to recall sequences in reverse order. The forward and backward spans are distinguishable and related differentially to math performance in children (Soltanlou et al., 2015).

Hoshi et al. (2000) revealed that backward span leads to greater activation in the bilateral prefrontal cortex than forward span. Therefore, the forward span in the letter span test represents the verbal WM, and the forward span in the Corsi block-tapping test represents visuospatial WM. For both forward and backward span of both verbal and visuospatial WMs, the score was the maximum sequence length at which at least two sequences were repeated correctly. The average of the backward spans of the two tests represents the central executive. Note that the backward span of the letter span test (e.g., Hadwin et al., 2005) and the backward span of the Corsi block-tapping test (e.g., Vandierendonck et al., 2004) have been separately reported as measures of the central executive. Vandierendonck et al. (2004) state a similar involvement of the central executive in the backward span of the Corsi block-tapping test and the backward letter/digit span (Vandierendonck et al., 1998). Moreover, according to the theoretical definition, the central executive is modality-independent (Baddeley, 1992) and is involved in manipulating both verbal and visual information. Therefore, the average of the backward span in the letter span and the Corsi block-tapping tests, which are functionally similar (Logie, 2014), was considered to be an indicator of the central executive in the current study. The internal consistency (Cronbach’s alpha) is 0.79 and 0.70–0.79 for the letter span (Kane et al., 2004) and the Corsi block-tapping test (Orsini, 1994), respectively.

#### Multiplication

In the present study, 16 simple and complex multiplication problems were used. Half of the problems of each set were used as trained problems and the other closely matched half were used as untrained problems. The sets were matched based on the sizes of the operands and results, as well as the parity of the operands and results, separately for simple and complex multiplication problems. The simple problems (e.g., 3 × 7) included two single-digit operands (range 2–9) with two-digit solutions (range 12–40). The complex problems included one two-digit operand (range 12–19) and one single-digit operand (range 3–8) with a two-digit solution (range 52–98). The sequence of small and large operands within the problems was counterbalanced. Problems with ones (e.g., 9 × 1), commutative pairs (e.g., 3 × 4 and 4 × 3) or ties (e.g., 6 × 6) were not used (for more see Soltanlou et al., 2018). According to the PET, which suggests the effect of math anxiety on complex tasks, and because of our small sample size, we only report the findings of complex multiplication problems. Trained and untrained multiplication task in the pre-training and post-training sessions has an internal consistency (Cronbach’s alpha) of 0.82 in the current study.

### Procedure

#### Measurement

This study is a part of a larger behavioral and neuroimaging project on math learning in children (Soltanlou et al., 2017, 2018). In a within-subject experiment, math performance of children was measured before and after training in both trained and untrained complex multiplication problems. The IQ, MAQ, and WM measures were administered after the post-training measure. Measurement of math anxiety after the math task has the advantage of avoiding any possible pre-judgment and bias about the forthcoming task in children (see also Ramirez et al., 2013). The math task was preceded by four practice trials. Problems were presented on a touch screen and children had to write their answers as quickly and accurately as possible and then in order to continue, they needed to click on a gray box presented on the right side of the screen (see Soltanlou et al., 2018 for more details). The written response was not visible to avoid any further corrections and to encourage children to calculate mentally. The problems of each set were presented in four blocks of 45 s, each followed by 20 s of rest. The sequence of blocks and problems within the blocks was pseudo-randomized. The problems, but not the sequence of the blocks or problems, were identical for each set in pre-training and post-training sessions. Whenever the total number of trials within a set was reached, the same problems were presented again after randomization. No feedback was given during the experiment. The design was self-paced with a limited response interval of 30 s for each problem. Therefore, due to inter-individual differences, the number of solved problems varied between children. The inter-trial interval was set to 0.5 s. The experiment was run using Presentation^®^software version 16.3 (Neurobehavioral Systems Inc.).

#### Training

Training was conducted via an online learning platform (Jung et al., 2015, 2016; Roesch et al., 2016), which allows for at-home training. The problems in the trained complex multiplication condition were randomly repeated six times in each training session. Each problem was individually presented along with 12 different choices including the correct solution (see Soltanlou et al., 2018). Response intervals of complex problems ranged randomly between 10 and 30 s, jittered by 2 s. Whenever the child did not respond within the response interval, the computer screen displayed the correct solution. Training was interactive because children had to compete with the computer. In order to create a more realistic competition, the computer responded incorrectly in 30% of the problems. To provide immediate feedback about the performance and to increase motivation, the scores of the child and computer were shown on the right side of the screen after choosing a solution. Both child and computer received one point for each correct answer and one point was deducted for each incorrect answer. The problem was presented until the child or computer responded correctly. Children were instructed to solve the problems as quickly and accurately as possible. Children performed seven sessions of approximately 25-min interactive training between two measurement times: one session in the lab and six sessions at home during about 2 weeks. The post-training session was conducted after these 2 weeks.

### Analysis

For the math task, the written responses by children were read out with the help of the RON program (Ploner, 2014). Response times (RTs) were defined as the time from problem presentation to pressing the gray box. Only mean RTs for correct responses (74.45% of problems across both measurement times) were included in the analyses. Error rate was defined as the proportion of incorrect or missing responses to the total number of presented trials. Furthermore, in order to approximate a normal distribution, an arcsine-square-root-transformation of error rate (Winer et al., 1971) was calculated. Thereafter, learning slopes were calculated by subtracting the mean RT and arcsine-square-root-transformed error rates of the pre-training session by post-training session separately for trained and untrained multiplication sets for each child. In both RT and error rate, larger values show higher training effects. Paired *t*-tests were conducted between trained and untrained sets for both RT and error rate learning slopes separately.

In order to test the associations between variables, correlation and regression analyses were calculated. Based on these analyses, mediation analysis was conducted by considering math anxiety as a predictor, learning slopes as dependent variables, and any WM component that significantly correlated with math anxiety, as a mediator (cf. Figure 1). According to Baron and Kenny’s (1986) causal-steps test (1986), four assumptions need to be met for mediation analysis (see also Field, 2013): (1) the total effect of a predictor on the dependent variable (path c) must be significant, (2) the effect of predictor on mediator (path a) must be significant, (3) the effect of mediator on dependent variable (path b), while controlled for predictor, must be significant, (4) the direct effect of predictor on dependent variable (path c’), while controlled for mediator, must be smaller than the total effect of predictor on dependent variable (path c) (cf. Figure 1). However, more liberal mediation tests such as the joint significance test (MacKinnon et al., 2002) suggest that only the second and third assumptions are required and the first and fourth assumptions are not necessary (for more see Fritz and MacKinnon, 2007). The Sobel test or delta method was used for the mediation analysis. This method estimates the standard error of the indirect effect and assumes the sampling distribution of the indirect effect as being normal^2^. It assesses the presence of mediation by dividing the indirect effect by the first-order delta-method standard error of the indirect effect and then compares it against a standard normal distribution. If the result of this calculation is significant, mediation is present (Fritz and MacKinnon, 2007). The analysis was completed using RStudio (RStudio Team, 2016) and jamovi software (jamovi project, 2018).

## Results

### Learning Slopes

A paired *t*-test on the RT learning slopes revealed a significant training effect in trained problems (*M* = 4.27 s, *SD* = 3.06 s) compared to untrained problems (*M* = 1.60 s, *SD* = 2.41 s), *t*(24) = 3.91, *p* < 0.001, showing that children responded faster to the trained set than untrained set due to training. A paired *t*-test on the error rate learning slopes again revealed a significant training effect in trained problems (*M* = 0.11, *SD* = 0.20) compared to untrained problems (*M* = -0.04, *SD* = 0.18), *t*(24) = 3.30, *p* = 0.003, showing that children made less errors when solving trained problems than untrained problems due to training.

### Correlation and Regression

The correlation and regression analyses revealed the following results. (1) No significant correlations between math anxiety and learning slopes (path c) were observed. (2) A negative correlation between math anxiety and visuospatial WM (path a) showed higher anxiety with decreasing visuospatial WM. Since math anxiety only correlated with visuospatial WM, further analyses were conducted only on this WM component.

Additionally, significant correlations between verbal WM and central executive, and between RT learning slope and error rate learning slope, were observed. No other significant correlations were observed (cf. Table 1).

**Table 1:** Correlation between math anxiety, WM components, and learning slopes.

			Correlations					
	Mean (SD)	Range	1	2	3	4	5	6
1. Math anxiety	12.92 (3.17)	0–20	–	–0.12	–0.43^∗^	0.02	–0.08	–0.31
2. Verbal WM	4.92 (1.04)	2–9		–	–0.14	0.35*	–0.10	0.15
3. Visuospatial WM	5.00 (0.58)	3–9			–	0.22	0.00	–0.20
4. Central executive	4.52 (0.81)	2.5–8.5				–	–0.12	0.17
5. RT learning slope	4.27 (3.06)	–					–	0.66**
6. Error rate learning slope	0.11 (0.20)	–						–


*N = 25, ^∗∗^*p* < 0.01, ^∗^*p* < .05, two-tailed.*

(3) Regression analysis to test the effect of visuospatial WM on error rate learning slope while controlling for math anxiety (path b) was only marginally significant, *R*^2^ = 0.23, *F*(2,22) = 3.30, *p* = 0.056 (cf. Table 2). The result revealed that the higher math anxiety and the higher visuospatial WM (but marginally significant) the lower math learning as indicated by error rates. This finding shows a suppression effect: while neither math anxiety nor visuospatial WM correlated with error rate learning slope, by inserting them together, they significantly predicted error rate learning slope. A suppression effect is defined when adding the third variable (i.e., WM) increases the effect of the independent variable (i.e., math anxiety) on the dependent variable (i.e., learning), which is the opposite effect of the third variable in mediation.

**Table 2:** Regression analyses (path *b*).

	*b*	*SE*	*B*	*t*	*p*
**Math anxiety and visuospatial WM → Error rate learning slope**					
Constant	1.19	0.45	0.00	2.64	0.015*
Math anxiety	–0.03	0.01	–0.48	–2.33	0.030*
Visuospatial WM	–0.14	0.07	–0.41	–1.98	0.060
**Math anxiety and visuospatial WM → RT learning slope**					
Constant	6888.40	7977.60	0.00	0.86	0.397
Math anxiety	–101.70	226.80	–0.11	–0.45	0.658
Visuospatial WM	–206.70	1246.90	–0.05	–0.21	0.836


*N = 25; *b*, unstandardized beta coefficient; *SE*, standard error of *b*; *B*, standardized beta coefficient. ^∗^*p* < 0.05.*

Regression analysis to test the effect of visuospatial WM on RT learning slope while controlling for math anxiety (path b) was not significant, *R*^2^ = 0.01, *F*(2,22) = 0.10, *p* = 0.905 (cf. Table 2). Since this assumption was not met for RT learning slope, further analysis was conducted only on error rate learning slope.

(4) The mediation analysis revealed that by inserting visuospatial WM as the mediator to the model, math anxiety significantly predicts (path c’) error rate learning slope (cf. Table 3). The suppression effect was also corroborated by this finding that the estimation of the total effect (path c) is closer to zero than the direct effect (path c’), and the estimation of direct and indirect effects have opposite signs (MacKinnon et al., 2000).

**Table 3:** Mediation analysis between math anxiety, visuospatial WM, and error rate learning slope.

				95% Confidence interval			
Effect	Label	*b*	*SE*	Lower	Upper	*Z*	*p*
Indirect	*a* ×*b*	0.011	0.007	–0.003	0.024	1.58	0.113
Direct	*c’*	–0.030	0.012	–0.053	–0.006	–2.48	0.013*
Total	*c*	–0.019	0.011	–0.042	0.004	–1.60	0.109


*N = 25; *b*, unstandardized beta coefficient; *SE*, standard error of *b.*^∗^*p* < 0.05.*

**FIGURE 2:**
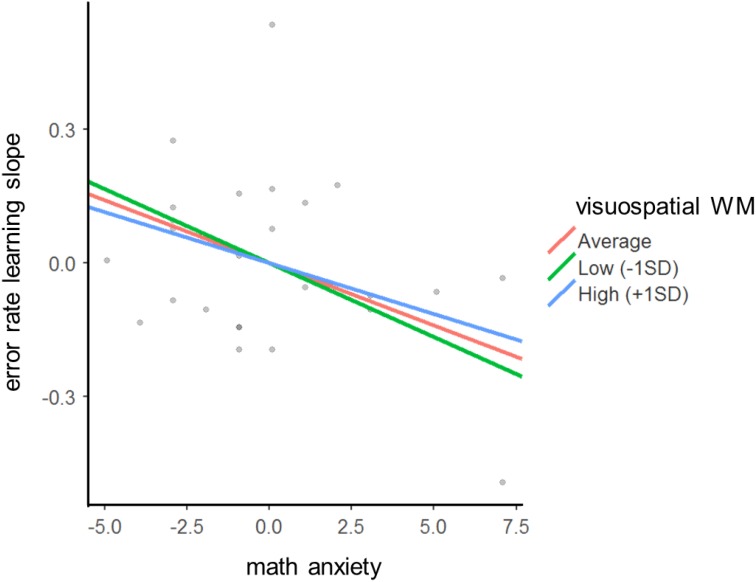
Simple slope analysis showing the effect of math anxiety on error rate learning slope at three different levels of visuospatial WM capacity. The scores of math anxiety and error rate learning slopes have been centered by subtracting each value from the mean. While children with low and average visuospatial WM capacity were significantly influenced by math anxiety and drew less benefit from multiplication learning, children with high visuospatial WM capacity were not significantly influenced by math anxiety.

In order to explore the relationship between these three variables, a simple slopes analysis (Aiken and West, 1991) was conducted on the z-transformed scores. According to the simple slopes analysis, the effect of math anxiety on error rate learning slope is investigated at low, average, and high levels of visuospatial WM capacity. As a standard method, low and high levels are defined as 1 *SD* below and above the mean, respectively. The analysis revealed that children with low (*b* = -0.03, *z* = -2.60, *p* = 0.009) and average (*b* = -0.03, *z* = -2.38, *p* = 0.017) visuospatial WM capacity were significantly influenced by math anxiety and got less benefit from multiplication learning (cf. Figure 2), while children with high visuospatial WM capacity are not significantly influenced by math anxiety (*b* = -0.02, *z* = -1.19, *p* = 0.233).

## Discussion

In the present intervention study, children improved after seven sessions of complex multiplication training. Moreover, an association between math anxiety, visuospatial WM, and math learning was observed.

We observed a significant negative relationship between math anxiety and visuospatial WM, suggesting that children with higher math anxiety have less storage capacity for visual and spatial information. This finding is in line with previous literature reporting the influence of math anxiety on visuospatial WM (e.g., Trezise and Reeve, 2018). Miller and Bichsel (2004) found math anxiety effects on visual WM but not on verbal WM. They suggest that while other types of anxiety affect verbal processes, math anxiety has a different unique effect on visual WM. In a similar way, Shackman et al. (2006) observed that anxiety selectively disrupts visuospatial WM but not verbal WM. However, the adverse effect of anxiety on other WM components has been shown as well. For instance, Hadwin et al. (2005) observed that low-anxious children aged 9–10 years old were faster in doing forward and backward digit span tasks (verbal WM and central executive) than high-anxious children, but not in a visuospatial WM task. Our finding suggests that because 5th graders rely on their visuospatial WM to solve multiplication problems, if math anxiety has any effect, this effect might be on this skill rather than verbal WM.

Although literature reported a strong association between WM and math performance (Aronen et al., 2005; Menon, 2016), we did not observe this relationship in the correlation analysis. However, visuospatial WM was a nearly significant predictor of error rate learning slope when we added math anxiety to the model. This finding might point to the necessity of math anxiety as an individual difference measure, which needs to be taken into account when we investigate math acquisition during development (Vukovic et al., 2013). As Vukovic et al. (2013) suggest, math anxiety influences how children utilize their WM capacity to learn math. The importance of visuospatial WM in multiplication problem solving has already been shown in children (Soltanlou et al., 2015, 2017). Unexpectedly, the relationship between visuospatial WM and error rate learning slope was negative, showing that children with higher visuospatial WM get less benefit out of multiplication learning. One interpretation might be because they had already few errors in pre-training, therefore, this short training did not lead to a significant improvement in these children. However, this association will be disambiguated later by exploring the interaction between math anxiety, visuospatial WM, and error rate learning slope.

Interestingly, by adding both math anxiety and visuospatial WM as predictors of math learning, a suppression effect was observed: the influence of math anxiety on math learning increased by adding visuospatial WM to the regression model. When exploring this relationship, we observed that while children with a low and average capacity of visuospatial WM are more influenced by math anxiety, children with a high visuospatial WM capacity can compensate the negative influence of math anxiety on learning. As Ashcraft and Kirk (2001) suggested, individuals with higher WM capacity have more resources to simultaneously deal with math anxiety and solve the math problems (see also Miller and Bichsel, 2004). The general pattern of findings – from the simple slope analysis – is partially in line with the study by Owens et al. (2012). They showed that trait anxiety is negatively correlated with cognitive performance in 12- to 14-year-old children with low WM capacity; however, no significant correlation was observed in children with average WM capacity. Contradictory to our findings, they found a positive relationship between trait anxiety and cognitive performance in children with high WM capacity.

It seems that the *combination* of high math anxiety and low WM is critical for hindering math learning. One might argue that children with high WM capacity have enough resources to attenuate the influence of math anxiety on math acquisition, which is in line with the PET. We suggest that this claim is correct if WM mediates the association between math anxiety and math learning, similar to several correlational studies. These studies revealed that either verbal WM (e.g., Owens et al., 2008) or visuospatial WM (e.g., Miller and Bichsel, 2004) mediates the anxiety-math performance association. There is a crucial conceptual difference between mediation and suppression: while WM reduces the influence of math anxiety on math performance in mediation, this effect increases in suppression^3^. So, while the correlational studies found the former, we observed the latter in our learning study. Furthermore, as Hopko et al. (2003) discussed, a single measure of math performance at a certain time is not purely a measure of competence, but a measure of both math anxiety and competence combined. Individuals start solving math problems with different levels of math anxiety, which is most probably represented in their output as well. We conclude that the findings of correlational studies may not be readily generalized to causal and intervention studies.

Furthermore, we found that math anxiety had a negative influence on children with low and average WM capacity but this influence was not significant in children with high WM capacity. As we explained in the introduction, there are two contradictory accounts of the relationship between math anxiety and WM capacity across the literature: one suggests that math anxiety has a negative impact in individuals with low WM capacity (Ashcraft and Kirk, 2001); the other suggests that individuals with higher WM capacity suffer more from math anxiety (Beilock and Carr, 2005). Our findings adhere to the first account, showing that children with higher WM capacity have enough resources to deal simultaneously with anxious thoughts and also store and manipulate new information (Eysenck et al., 2007). As Lee and Bull (2016) argued, WM is needed when learning new academic skills to integrate the new information with previously acquired knowledge. This explanation is corroborated by neuroimaging studies revealing increased prefrontal activation for emotion regulation, in addition to the fundamental role of the right amygdala in emotion processing (Young et al., 2012). Therefore, prefrontal capacity that subserves cognitive processes such as WM is partially allocated to regulate these affective responses. Hence, this capacity is less available for the cognitive task at hand, such as solving a math problem (Eysenck and Calvo, 1992; Eysenck et al., 2007). Therefore, it is reasonable to see a stronger association between math anxiety and math learning in children with lower WM capacity.

Inconsistent with the PET, performance effectiveness (response accuracy) and not processing efficiency (response time) was influenced by math anxiety in our intervention study. The prediction of the PET has received support and contradictory evidence in the field of numerical cognition. For instance, Ng and Lee (2010) observed that processing efficiency – but not performance effectiveness – on a mental arithmetic task is affected by test anxiety in 10-year-old children. Vukovic et al. (2013), however, observed a negative correlation between math anxiety and performance effectiveness in their longitudinal study, which supports our findings (see also Devine et al., 2012). Nonetheless, they did not measure the response time in their math tasks, which might have shown a significant association as well. In line with their finding, Trezise and Reeve (2018) showed that while anxiety is negatively related to the response accuracy in two low- and high-time pressure conditions, there is no significant correlation between math anxiety and response time in 14-year-old children. It seems that the underlying mechanisms of one-time math performance measures differ from math learning. We suggest that – in line with the PET – a negative correlation between math anxiety and math learning was observed in the present study; however, contradictory to its prediction, this relationship was between anxiety and response accuracy, and not response time.

### Limitations

There are some limitations that need to be taken into account for interpretation of our findings which should be addressed in future studies. Our study was a complex and effortful intervention study, in which not so many children can be easily tested, as compared to cross-sectional correlational designs. Therefore, null effects in particular were and should be interpreted with caution due to low power. Especially, if there are smaller intervention or mediation effect sizes, it is conceivable that they might be observed in a larger sample. Moreover, in order to reduce confounding effect of maturation and education, we conducted this study in a group of 5th graders with a limited age range. Therefore, the influence of math anxiety on learning, which we observed here, needs to be further investigated in larger samples and in different age groups to see whether our findings can be replicated and generalized.

Moreover, it is suggested to measure the other types of anxieties to see whether our findings are math specific or related to trait or test anxiety as well. Although we investigated several other interesting factors such as gender, task complexity, and self-attitude in our study, however, because of the small sample size, we focus only on the most important question: whether math anxiety influence on math learning in children. Therefore, it is suggested for future studies to consider these factors as well.

## Conclusion

Most studies so far have only investigated the influence of math anxiety and WM on math performance. In such studies, both variables have a negative impact on math performance, and in some studies (in line with the PET) WM mediates the influence of math anxiety on math performance.

Our study suggests that the case might be different for the influence of math anxiety and WM on *math learning*. While an influence of WM on math performance is ubiquitous, we failed to find a significant influence of any of the WM components on math learning. This might be partially consistent with a recent meta-analysis showing that WM training does not transfer strongly to other skills and capabilities like math (Melby-Lervag et al., 2016). So, if a child has a higher WM capacity or even if WM is improved after training, he might have a good math performance – in both pre- and post-training measures – but not necessarily improves dramatically after math learning as compared to pre-training performance.

While WM might not predict math learning *per se*, it fosters the influence of math anxiety on math learning. Children with a low visuospatial WM capacity suffer most from math anxiety when they have to learn math. The explanation for this is in line with the PET. If children have no or little math anxiety, enough WM resources are left for math learning, so no major problems occur. If they have high math anxiety and high visuospatial WM, some WM resources are needed to deal with math anxiety but learning is still supported. However, if they have high math anxiety and low visuospatial WM capacity, math learning is significantly impaired. These children have less capacity to learn new math contents because they need all the resources to deal with their math anxiety. This finding might be helpful for future interventions and suggests that in order to improve children’s performance, both math anxiety and WM capacity need to be considered.

Our findings show that math anxiety plays a major role in multiplication learning and that data from performance studiescannot be readily generalized to learning studies. However, multiplication learning is a rather easy task (even if the problems are difficult). The picture might change for other math content. Our study suggests that it is worthwhile to examine the influence of math learning in other math areas as well. After all, learning math is what all children are asked to achieve and where many children suffer tremendously. Therefore, although intervention studies are hard to conduct, we believe it is a worthy and necessary effort to be addressed in future studies if we want to understand and promote math learning in children.

## Author Contributions

All authors designed and conceptualized the study. MS and CA collected the data. MS analyzed the data and wrote the main manuscript text. All authors reviewed the manuscript.

## Conflict of Interest Statement

The authors declare that the research was conducted in the absence of any commercial or financial relationships that could be construed as a potential conflict of interest.

## Footnotes

1 Krinzinger and colleagues (one of which – Nuerk – is co-author both of her and our paper), developed the German version of the MAQ, which we used in our study. Their results strongly suggested that the two subscales of “How happy or unhappy are you if you have problems with...?” and “How worried are you if you have problems with...?” actually measure the same construct, which is negative emotions and anxiety concerning mathematics (see also Krinzinger et al., 2009). Krinzinger et al. (2007) observed a correlation of 0.78 between these two constructs in third graders, which is close to reliability. Since this means that we essentially measure the same construct twice, we only used the first subscale in the present study. As this study is a part of a larger project of math training in children, we had to shorten some tests by only concentrating on the most relevant parts. Therefore, these two MAQ subscales, which essentially measured the same construct in German children, were the natural candidates for that.

2 Note that the non-parametric percentile bootstrap confidence interval method with 5000 samples, which does not assume a normal distribution of the indirect effect, revealed a similar suppression effect in mediation analysis (see the Result section).

3 Note that this relationship was not a moderation because (1) in moderation the relationship between predictor and dependent variable is significant *per se* but it changes by the third variable, however, this relationship was not significant in our data, (2) the interaction of math anxiety and visuospatial WM (moderation analysis) did not significantly predict learning slopes.
